# The place of public inquiries in shaping New Zealand's national mental health policy 1858–1996

**DOI:** 10.1186/1743-8462-2-24

**Published:** 2005-10-10

**Authors:** Warwick Brunton

**Affiliations:** 1 Department of Preventive and Social Medicine, University of Otago, P.O. Box 913, Dunedin, New Zealand

## Abstract

**Background:**

This paper discusses the role of public inquiries as an instrument of public policy-making in New Zealand, using mental health as a case study. The main part of the paper analyses the processes and outcomes of five general inquiries into the state of New Zealand's mental health services that were held between 1858 and 1996.

**Results:**

The membership, form, style and processes used by public inquiries have all changed over time in line with constitutional and social trends. So has the extent of public participation. The records of five inquiries provide periodic snapshots of a system bedevilled by long-standing problems such as unacceptable standards, under-resourcing, and poor co-ordination. Demands for an investigation no less than the reports and recommendations of public inquiries have been the catalyst of some important policy changes, if not immediately, then by creating a climate of opinion that supported later change. Inquiries played a significant role in establishing lunatic asylums, in shaping the structure of mental health legislation, establishing and maintaining a national mental health bureaucracy within the machinery of government, and in paving the way for deinstitutionalisation. Ministers and their departmental advisers have mediated this contribution.

**Conclusion:**

Public inquiries have helped shape New Zealand's mental health policy, both directly and indirectly, at different stages of evolution. In both its advisory and investigative forms, the public inquiry remains an important tool of public administration. The inquiry/cause and policy/effect relationship is not necessarily immediate but may facilitate changes in public opinion with corresponding policy outcomes long after any direct causal link could be determined. When considered from that long-term perspective, the five inquiries can be linked to several significant and long-term contributions to mental health policy in New Zealand.

## Background

New Zealand's mental health policy and services have evolved along similar lines to other "old Commonwealth" countries. Like other British colonies of settlement in the nineteenth century, New Zealand endeavoured to adopt the progressive ideas of the non-restraint system of care and treatment, along with its corollaries of special purpose residential institutions, medical management and separate legislation. Colonial administrators and politicians preferred the English system of institutional care over the Scottish mixed system of lunatic asylums and boarding out or community care. The concentration of specialised resources in residential care facilities that were known at different times as lunatic asylums, mental hospitals or psychiatric hospitals isolated them professionally, socially and administratively from mainstream health and social services. Institutions remained dominant until the late twentieth century, even though the spectrum of mental health services widened in response to demands for general hospital psychiatry from the 1920s, community care from the 1960s and, more recently, deinstitutionalization. The ebbs and flow of policy and service development in New Zealand, however, as in many other countries, typify a classic feature of mental health policy – alternating bursts of policy development and funding interspersed with long periods of quiet incremental change, indifference, or even stagnation. These longstanding cycles make mental health an interesting policy domain in which to study the impact of public inquiries as an instrument of reform.

A public inquiry is a relatively independent investigation that considers facts and evidence on some assigned topic, draws conclusions, and reports, usually with recommendations. An inquiry is publicly accountable to the New Zealand Parliament or to the executive branch of government, most usually the Governor-General (the Queen's representative), government, minister, or some senior official, according to the status of the inquiry.

The public inquiry is a very versatile tool of public administration and remains part of the apparatus of government in various Commonwealth jurisdictions. There has been a distinct shift from inquiries by parliamentary select committees towards executive-appointed inquiries since mid-Victorian times as the device has evolved within the Westminster constitutional system.[1-3] The executive-appointed inquiry was legitimated in New Zealand in 1867 and is now the basis of most inquiries. Such inquiries can be initiated by the government, (royal commissions and commissions of inquiry), ministers of the Crown (committees of inquiry), statutory agencies or officials. Royal commissions are generally held to have greater prestige and standing than other forms of investigation, and they have been used traditionally to inquire into topics of outstanding public importance. Royal commissions are of medieval origin. The Governor-General of New Zealand appoints them in the Queen's name. Commissions of inquiry are constituted by an Order in Council under the Commissions of Inquiry Act 1908. The Governor-General acts on the advice of ministers in making appointments to either type of inquiry. Many Acts of Parliament vest statutory bodies with the powers of a commission of inquiry.

The Commissions of Inquiry Act 1908 and amendments give a properly constituted inquiry wide powers to conduct proceedings, summon witnesses, call and take evidence on oath, inspect and examine documents, or to require them to be produced. Some modern inquiries have undertaken or commissioned their own research. The cost of an inquiry depends upon its duration, the number of members, the size of the secretariat, the amount of travel for hearings or site visits, or the use of overseas experts, counsel and technical advisers. A very comprehensive manual – the latest procedural guidance to New Zealand inquiries prepared since 1925 – is mindful of the growing body of precedent and inquiry-related jurisprudence.[4]

Some scholars in the United Kingdom, Australia, Canada and New Zealand have been interested in the general policy-making role of inquiries. Theses by Alan Simpson (1972) and Deborah Iversen (1992) are the best general source of information about public inquiries in New Zealand. Their research shows an enormous range of topics considered by inquiries. Published checklists do not include every public inquiry held in New Zealand.[5,6]

Some newsworthy incident, revelation, or a level of public disquiet that makes an issue politically sensitive usually triggers an inquiry. Setting up an inquiry allows for an issue to be carefully considered for a time without disrupting the regular administration of government. An inquiry has prestige, legal authority to obtain information and it can provide some protection to parties. Membership can be determined according to the nature of the issue. In some cases, representatives of particular interest groups might be brought together to facilitate consensus-building; in others, respected arbiters may be needed. Much of an inquiry's work is publicly visible. The invitational and participative approach of a modern inquiry widens the input to government policy-making.

An inquiry is relatively free within certain parameters to interpret its mandate, determine how it will gather information, formulate its arguments, organise its report, and make recommendations as it chooses. The membership, terms of reference, and duration of an inquiry – all of which are determined by the minister or government of the day – affect the degree of independence.

Ad hoc and temporary, an inquiry is said by Hallett to arise suddenly, like a mushroom and to vanish just as suddenly, once it has presented or forwarded its report to the initiating public body or official.[7] An inquiry's report is then analysed by officials and ministers, who decide how to handle the recommendations. The report is usually published. A number have been tabled and printed in the parliamentary papers.

A public inquiry serves an advisory or investigative function. An advisory inquiry addresses a broad issue of public policy. An investigatory inquiry establishes the facts of some incident or tragedy. The distinction between advisory and investigative inquiries is not always clear-cut. Investigation of an incident may lead to consideration of the policy context. The study of policy may expose abuses or mistakes.[8,9] Simpson claims that the great majority of inquiries in New Zealand have been investigative.[10] That is certainly so for mental health inquiries. Significant examples of investigative inquiries in this field include inquiries into abuse and ill-treatment at the Wellington Lunatic Asylum (1881), structural defects and faulty workmanship at Seacliff Lunatic Asylum (1888), suicide at Ashburn Hall licensed house (1896), a tragic fire at Seacliff Mental Hospital (1943), short-staffing in mental hospitals (1946), the administration of Oakley Hospital, Auckland (1971), the state of forensic psychiatry services (1987), and the death of a person being taken into psychiatric care (1994). My analysis of mental health inquiries held between 1987 and 1995 showed that these were also investigative. Investigative inquiries provide a sort of 'ritual cleansing' as a former New Zealand top mental health official once put it.[11] On the other hand, the spate of investigatory mental health inquiries in recent years has raised hints about the futility of 'another cycle of Inquiry [sic] fatigue,' a standpoint warranted, perhaps, by mixed evidence about the outcomes of earlier investigative inquiries.[12]

### New Zealand's general mental health inquiries

Five general or policy-advisory inquiries have been conducted into the state of mental health services (as that term was understood at the time) since the institution of parliamentary government in New Zealand in 1854. They were the

• Select Committee of the House of Representatives on a General Lunatic Asylum (1858),

• Joint Parliamentary Committee on Lunatic Asylums (1871),

• Board of Health Committee on Psychiatric Services in Public Hospitals (1957–60),

• Royal Commission on Hospital and Related Services (1972–3), and the

• [Ministerial] Inquiry in respect of Certain Mental Health Services (1995–6).

The full titles encompass the main forms of inquiry available under New Zealand law. These inquiries will be referred to hereafter by their abbreviated title. The nature and number of advisory mental health inquiries can be confidently established from a study of published official records. A sixth, the Committee of Inquiry into Mental Defectives and Sex Offenders (1924–25), would have been included had it interpreted 'mental defective' to include mental illness as well as what is nowadays usually called intellectual handicap or intellectual disability. Some other advisory inquiries have touched incidentally on mental health matters, e.g., those set up to investigate law and order, social security, or government administration. Such inquiries are not included in this study.

## Methods

The study was undertaken by reviewing the literature on the evolution, functioning and achievements of public inquiries as an instrument of government policy-making in New Zealand and comparable Commonwealth jurisdictions. The research on the five mental health inquiries used standard historical method based on published and unpublished official records. The *New Zealand Parliamentary Debates *provided the political context of each inquiry. The reports of three inquiries were published in the *Appendices to the Journals of the House of Representatives *and the others were published separately by the Department/Ministry of Health. Terms of reference and membership of some public inquiries were published among the official notices of government in the *New Zealand Gazette*. Records from the five inquiries have survived among the archives of the Legislative Department, the Department of Internal Affairs (which has general responsibility for the administration of public inquiries), and the departments of state responsible at different times for national mental health policy: the Lunatic Asylums (1876–1905)/Mental Hospitals (1905–1947) Department, and the Department (1947–1992)/Ministry (since 1992) of Health. The Division of Mental Hygiene or Mental Health (1947–1992) provided a particular focus within the Department of Health, as has the Mental Health Section or Directorate in the Ministry of Health. The records and reports of each inquiry provide rich sources of material. Unpublished records of an inquiry may include the warrant of appointment, a transcript of proceedings, manuscript and printed reports, and administrative and financial correspondence. Official records have been augmented by reference to contemporary press comment. Archives of the Legislative Department and Internal Affairs Departments and those of some provincial governments were accessed to provide information on the follow-up to the inquiries of 1858 and 1871. The Department of Health Archives were used to obtain comprehensive information about the extent of bureaucratic influence over later inquiries. Records of the Ministry of Health concerning the Ministerial Inquiry of 1995–6 have not used because of the likely sensitivity and topicality of those events.

## Results

In this section, I will outline the background of each of the five inquiries, the membership, duration, procedures, and outcome. I will then consider how each inquiry contributed to national mental health policy.

### Select Committee (1858)

Insanity was scarcely anticipated as a public policy matter when New Zealand became a British colony in 1840. The earliest known insane persons came to official attention because they posed a threat to public safety, could not safely look after themselves, or because they had no family to care for them. Care of such persons fitted the generally accepted purposes of colonial government and the civilising mission of British imperialism. The foundation documents of the new colony anticipated a system of law and order that was gradually established in the principal Pakeha (European immigrant) settlements. The colony's first mental health legislation, the Lunatics Ordinance 1846, limited disposition of a lunatic to a gaol, house of correction, or public hospital until the person was discharged or transferred to a 'public colonial lunatic asylum'. Lunatics who were not dangerous were a secondary concern. This pragmatic approach was typical of other British colonies, but it had its drawbacks. Opinion-makers considered improvised care in gaols or public hospitals as inhumane, inadequate and administratively irksome. A 'community care' option of boarding out or repatriation was rarely used, except perhaps for Maori.

Colonial authorities took their lead from the imperium where a burst of activity had consolidated a policy framework in England during the 1840s. The state regulated admissions and discharges through a process now known as committal. Marketplace activities were publicly monitored. A chain of public lunatic asylums was under construction across the country. A specialised bureaucracy was set up to inspect facilities and to set standards. This framework was gradually adapted to the circumstances of constituent parts of the realm and of the settler colonies. Given the cultural background of the British settlers, in essence, the choice lay between the English model of lunatic asylums or the Scottish mixed model of institutions and grant-aided boarding out of suitable patients with private families. In the ensuing debate, the ideals of a properly designed lunatic asylum were invariably upheld, complete with a system of patient classification, a safe, healthy and orderly environment, and a regime of moral management, which included minimal physical restraint.

By 1858, New Zealand politicians favoured the English system of institutional care over the Scottish mixed model. The systemic policy issue to be investigated was the best way to provide a proper lunatic asylum. The ideal was widely respected but of the eight provinces, only Wellington had actually built such an institution by 1858. With the small number of lunatics in each province and with a national total of 50–100 chronic lunatics (depending on whether official returns or political estimates were used), the idea of a central asylum for the whole colony made sense to provincial politicians on the grounds of humanity and efficiency. The financial argument – that a colonial facility might relieve the provinces of financial responsibility – hinted at the underlying political dynamic. The provincial system (1852–76) created inherent tensions between the relative responsibilities, authority and fiscal powers of provincial governments and the central government. Provincialism was intended to address demands for a measure of self-government in the isolated and scattered settlements of the colony. Financially struggling provinces, however, quickly learned how hard it was to discharge their responsibility to provide adequate and acceptable care for lunatics. In such circumstances, transferring responsibility to the central government was attractive to the provinces.

The Select Committee's membership reflected this political dynamic. Four of the six provinces were represented on the 8-person committee. Every member had been involved in provincial politics and five held concurrently parliamentary and provincial seats. EW (later Sir Edward) Stafford, for example, was both a provincial and national politician and took a lead as a member of the Select Committee. As Premier and Colonial Secretary (the minister nominally responsible for lunacy policy and legislation), he personally followed up the Select Committee's report.[13] Only two committee members – one a magistrate and the other a medical practitioner – were professionally acquainted with the problem of lunacy.

The Select Committee heard evidence from medical practitioners and from those in charge of the local gaol and hospital at Auckland, the then capital. Politicians with some knowledge of the situation in other provinces were also examined. The Select Committee used a standard form of questioning and it kept an unpublished verbatim record of evidence by the 14 selected witnesses.[14] The Select Committee took two and one-half months to complete its work and submitted a two page report.

Although the Select Committee was apprised of the Scottish model, the weight of evidence favoured an institutional approach. Some of the six recommendations (see Table 1) embodied the contemporary ideals of a lunatic asylum, purpose-built for the non-restraint system that was a hallmark of leading English asylums. A comprehensive but stand-alone legal framework for lunacy was also recommended. The Stafford government adopted these ideas although they were not immediately implemented.[15-18] After a change of government in 1861, planning for the central asylum ceased in favour of the establishment of a network of provincial asylums. When Stafford again became the Premier (1865–9), he astutely upheld that decentralised approach.[19] The central government retained legislative responsibility, set a few standards, and published the reports of provincial inspectors, but was otherwise reluctant to interfere in what had become by then a recognised provincial domain.

**Table 1:** Implementation of General Mental Health Inquiry Recommendations, 1858–1996

INQUIRY	KEY RECOMMENDATIONS	IMPLEMENTATION
**Select Committee (1858)**		
	Establish colonial lunatic asylum.	Accepted 1858 but not implemented.
	Appoint commissioner to choose site.	Implemented 1858.
	Obtain expert advice on asylum design and organisation.	Implemented 1858.
	Adopt comprehensive and liberal treatment in asylum.	Accepted and applied variably by provinces.
	Amend lunacy law.	Implemented 1858.
	Revise lunacy law entirely.	Implemented 1867–8.
**Joint Committee (1871)**		
	General government to ensure proper provision for lunatics where provision inadequate.	Limited to guidelines before 1876 and direct management of asylums afterwards.
	Appoint specialist psychiatrist to supervise and control all asylums.	Implemented 1876.
	Obtain more information before making decision about central asylum.	Implemented 1872–4.
	Improve all asylums, especially Karori (Wellington).	Left to provincial governments.
**Board of Health Committee (1957–60)**		
	Increase general hospital acute psychiatric beds in four main cities immediately.	Implemented progressively under hospital capital works programme.
	Develop regional psychiatric units and outpatient clinics in 6 other cities.	Implemented – first unit opened 1963. Divisional outpatient services expanded as staffing permitted.
	Establish staff in the units as per staff: patient ratios.	[Implemented].
	Establish child psychiatry units in four main centres when staff available.	Adopted but implemented through child health clinics.
	Intensify specialist staff recruitment.	Ongoing implementation.
	Second mental hospital staff to psychiatric units.	Implemented for first units then phased out.
	Improve public attitudes towards mental illness.	Accepted. Intensified public relations with World Mental Health Year 1960.
	Relax legal restrictions on patients' personal rights.	Implemented 1961.
**Royal Commission (1972–3)**		
***First report***	Continue mental health lead by way of allowance.	Adapted.
	Improve psychiatric hospital staffing to eliminate need for pay differential.	Accepted.
	Set up independent study of poor working conditions that affect staff shortages.	Adapted then rejected 1975.
	Review differential conditions of employment.	Implemented.
	Review entry requirements to encourage recruitment of male psychiatric nurses.	Reviewed for more consideration.
	Study extent of recruitment problem.	Adapted then rejected 1975.
***Second report***	Establish national Institute of Psychiatry.	Referred for consideration by key agencies.
	Advise those concerned without delay.	Implemented.
***Third report***	Undertake national survey of service needs of mentally handicapped patients in psychiatric hospitals.	Implemented 1973–4.
	Progressively move multiple-handicapped patients to general hospital care.	Deferred pending survey results.
	Care for dual diagnosis or behaviourally disturbed mentally handicapped patients in general or psychiatric hospitals.	Deferred pending survey results.
	Develop appropriate placements at home in foster home, community house or small special purpose institution.	Deferred pending survey results.
	Discontinue practice of placing mentally handicapped patients in psychiatric hospitals.	Deferred pending survey results.
	Transfer responsibility for mental handicap services from Health to Social Welfare Department.	Adapted for inter-departmental consultation.
	Place moratorium on psychopaedic hospital development.	Implemented 1973.
	Discontinue hospital model of care for mentally handicapped.	Addressed through national needs survey.
	Actively promote measures to prevent mental handicap.	Required further investigation.
	Teach medical students modern views on management of mental handicap.	Required further investigation.
	Urgently support home care, IHC facilities, small homes and hostels under national plan.	Accepted in part but subject to needs survey results.
**Ministerial Inquiry (1995–6)**	Inquiry team should monitor implementation of its recommendations.	Rejected.
	Increase mental health funding between $125–140 M. over 5 years.	Adapted.
	Ring-fence mental health funding.	Rejected.
	Establish Mental Health Commission and National Advisory Board.	Adapted.
	Request MHC to prepare national blueprint for mental health services.	Implemented 1998.

### Joint Committee (1871)

By 1871, most provinces had built their own public lunatic asylum or were well on the way to doing so. Asylums in the gold-rich provinces achieved very creditable standards, but slower developing and cash-strapped provinces lagged behind. Only the smallest provinces still relied on makeshifts or made extra-territorial arrangements with asylums in other provinces. From the late 1860s, asylum standards were sucked into wider political moves towards nationally uniform standards and policies developed and monitored by new departments of the central government. Provincialism was waning although it remained a potent force in some parts of the colony, hence representation of all save the two smallest provinces on the Joint Committee.

The Legislative Council (upper house) established this inquiry because this body took a consensual though elitist interest in lunatics as one group in society who carried no political weight.[20] Dr A Buchanan, MLC (Otago Province), a key proponent of lunacy reform and chairman of the Joint Committee, believed that less than half-a-dozen parliamentarians held the cause of lunacy reform 'earnestly at heart'. Most of those were members of the Legislative Council. Four were medically qualified, and another lay member was the Inspector of Asylums in Otago. These members took a very active role in the work of the Committee, and spoke authoritatively and knowledgeably about progressive practice from their personal visits to provincial and English asylums.[21-23] Buchanan could not find the same enthusiasm among members of the House of Representatives (lower house). Joint Committee members from the Legislative Council contributed strongly to parliamentary debates on the topic, but committee members from the lower house did not show the same zeal. The mover was the only member who participated, and even he showed little sustained interest.[24] Buchanan was upset by the indifference of elected representatives whom, he said, would let the weakest sections of society go to 'the wall' for the sake of retrenchment.[25] He shared his opinions freely and forcibly with an eminent Scottish psychiatrist:

We are willing enough to 'go in' for reproductive Public Works. But, of course, the care of the Insane costs money, and does not 'pay.' [26]

Like the Select Committee, the Joint Committee had to meet and complete its work during the short parliamentary session that typically lasted three months. In five weeks, the Joint Committee heard from five invited witnesses, four of them local asylum doctors. The Joint Committee submitted the briefest report: one sentence criticised the current situation and four more set out the recommendations. The transcript of evidence was published as an attachment.[27]

The Joint Committee called for improvement in all asylums, and looked to the general government to make proper provision for lunatics through the appointment of a specialist psychiatrist who would supervise and control all lunatic asylums. The Joint Committee also recommended that more information be obtained before a decision was made about whether or not to proceed with a central asylum for the colony. The recommendations were adopted by the Legislative Council, but not by the House.[28,29] The government stalled. Expert advice was sought from Dr E Paley, Victoria's Inspector of Asylums, who visited New Zealand asylums in 1872.[30] Paley rejected the central asylum, but supported the idea of a specialised national inspectorate. He went further by suggesting that resident medical superintendents should be appointed to run larger asylums and that they should have a proper complement of attendants based on a staffing formula.[31] Paley's report was not published until 1874, the same year that financial provision was first made for the national inspectorate.[32] The inspector's job was advertised in Britain in 1875 and the appointee, Dr FWA Skae, took up his duties as first head of the Lunatic Asylums Department in July 1876.[33,34]

Skae's appointment reflected the government's decision that lunacy administration should revert to the central government in the redistribution of government activity when the provincial administrations were abolished in 1876. A department of state was set up to advise ministers on policy, to run and inspect public asylums, and later to regulate the country's only licensed house (private mental hospital). Under a near century of departmental administration until 1972, the country's psychiatric services slowly became characterised by national uniformity, a virtual bureaucratic monopoly, separatism from mainstream health services, and a predominantly institutional mind-set. These features were compounded by fickle public attitudes and the low political-fiscal priority given to mental health. Multiple pressures of overcrowding, large numbers of long-term residents, and severe staff shortages persisted long after a general therapeutic despair disappeared with the advent of electro-convulsive therapy and tranquillisers in the 1940s and 1950s.

Institutions were the cornerstone of psychiatric care and treatment in the New Zealand throughout this period, as they were in western countries generally. Institutions expanded in size thanks to elastic social and medical definitions of insanity. The number of institutions also grew as rail and road systems opened up new regions to settlement. After years of discussion, the name mental hospital was officially adopted in 1905 to replace the outmoded nomenclature of lunatic asylums. In its fullest sense, the specialised mental hospital came to incorporate 'reception homes' and other residential facilities for early treatment (after 1898) which patients were encouraged to enter voluntarily following a law change in 1911. Villa-style accommodation for long-term patients, which aspired to regain the rustic appeal and quasi-domestic scale of early asylum ideals, was another feature of the mental hospital. The villa principle was intrinsic to all new institutions from 1903. Community amenities like halls, libraries, canteens, and sports fields, were later additions. The mental hospital was also identified with specialists in psychiatry, and a cadre of professionally trained mental nurses rather than attendants. The new model attempted to capture the therapeutic glamour and favourable public image of general hospitals, which were run by ad hoc local authorities known as hospital boards.

Progress towards these lofty ideals was halting, patchy and a reflection on the low political priority of mental health. A significant credibility gap emerged between the ideals and the realities of overcrowded and short-staffed facilities. Outmoded design features and environments of those Victorian era institutions that were built in permanent materials hampered progress towards more modern approaches. A general inquiry was called for in 1903  when the overall policy of a long-serving departmental head seemed outdated. [35] Although the government successfully resisted the pressure, reforms were expedited. A deputy inspector-general was appointed, and that gave fresh impetus to reformist policies. That deputy, who was promoted to run the Department in 1907, himself became stale and tired by 1925. His dogged pursuit of a policy of mental hospitalisation did not match post-war pressure for a fresh approach based on intermediate facilities that would avoid the stigma of mental hospitals. Pressure for an inquiry in 1925 was only averted by a package of overdue reforms, including the establishment of the first outpatient services and observation wards in general hospitals.[36]

The establishment and extension of outreach services, however, fluctuated and was very haphazard. Progress was dependent upon the initiative and priority of local hospital boards and the availability of hard-pressed institutional psychiatrists, whose first priority had to be to mental hospital patients committed to their charge. Under the first Labour Government's Social Security Act 1938, the full cost of care and treatment in mental hospitals (1939) and general hospitals (1941), among other health benefits, was met from taxation rather than patient fees. Public expectations of the public hospital system were heightened by the ideals of that legislation, by significant progress in biomedical technology, and years by post-war prosperity. The financial effects of these changes foreshadowed the government's assumption of national capital planning procedures and the full cost of operating all public hospitals in 1957.

### Board of Health Committee (1958–60)

By the 1950s, the patchy growth of general hospital psychiatry was less tolerable to informed opinion in some hospital boards and the press. The groundswell of criticism was sufficient to prompt the next inquiry.[37-40] Elected politicians were receptive to this groundswell, but the real political actors were the health bureaucrats. Managed devolution was their agenda: how to expand peripheral services without eclipsing the paramountcy of institutional services.

As a technical/advisory committee, the Board of Health Committee was the only inquiry with a very high level of professional input. The committee's membership was stacked with senior health officials and long-standing hospital board politicians. This mix was probably designed to anticipate and manage one member, Dr W. Ironside, an academic psychiatrist whose views on general hospital psychiatric services and curbs on mental hospital growth were well in advance of prevailing official opinion. This member seems to have negotiated the right to record his views separately in a personal submission, which was included in the committee's report. This device made for a unanimous report, but it also provides a tantalising glimpse of tensions within the committee, which had trouble getting its members together. Internal dissent delayed completion of the 27-page report. The Minister's comments took 9 months to appear.[41]

The Board of Health Committee allowed 'certain national bodies and organisations interested in this matter' to make submissions, a procedure that effectively managed debate without exposing official institutional orthodoxy to the ideologies of pressure groups [42]. The committee was 'particularly impressed' with the opinions of the British Medical Association and the medical superintendents of general and mental hospitals, who had been sent a questionnaire by the committee.[43]

Departmental officers drafted the report and completely dominated a board sub-committee that rearranged the final recommendations.[44] These called for an immediate increase in the number of acute psychiatric beds in the four metropolitan general hospitals and the development of inpatient and outpatient services in six other cities. These units were to be staffed seconded from mental hospitals according to appropriate staff-patient ratios. The report recommended the establishment of specialised child psychiatry units in the four main cities when staff were available. Other recommendations were of a more general nature, for example, increased recruitment of specialist staff, improved public attitudes and a relaxation of restrictions on patients' personal rights.

The main recommendations accorded with departmental interests and were shaped by officials. Ironside's ideas were sidelined because the committee accepted a major and ongoing role for departmental institutions, which was the long-held view of the Department's Mental Hygiene Division. The Division could therefore accept general hospital units provided that the Division staffed them.[45] Implementation was reasonably successful although it took some years to fully implement some ideas.

### Royal Commission (1972–3)

The threat to institutional dominance in the spectrum of psychiatric services was compounded after 1965 by moves to integrate the administration of psychiatric and general public hospitals under local hospital boards. This culminated in industrial action by institutionally-based psychiatric nurses, who were apprehensive of the possible effects of the imminent transfer upon their pay and working conditions. Local management difficulties at Oakley Hospital, Auckland, compounded the uncertainty and led to a commission of inquiry in 1971. Meanwhile, the Public Service Association, the staff union, pressed for a public inquiry into mental health services generally.

The Department resisted this pressure and recommended a wider and three-stage inquiry after the Medical Association of New Zealand (formerly the New Zealand Branch of the British Medical Association) lobbied the Minister. Terms of reference were cobbled together from a contemporary Canadian royal commission.[46] The full title of the Royal Commission consciously echoed that of a major departmental policy statement, *A Review of Hospital and Related Services *(1969), which was premised on the notion of integrated health care administration. The Royal Commission was one of a number set up by the weary National government, which had been in power since 1960, in its final term of office (1969–72). The political timeframe shelved a hot industrial issue well beyond the dates of the proposed transfer and the 1972 general election.

The government caucus was consulted before Cabinet made a final decision on the membership of the Royal Commission whose composition offers an interesting insight into the trade-offs among experience, skills, objectivity and outlook sought by the Government [47]. Sir Keith Holyoake, the then Prime Minister (1960–72), said of the Royal Commission:

You look round to find some psychiatric bloke – he would be biased in one way or the other. As far as he could see – and this was not for the record or for quoting – he would rather have the best laymen available.[48]

The appointment of a distinguished lawyer or judge, in this case, a Queen's Counsel, to head the inquiry, followed convention. In keeping with another general trend, previous experience as an inquiry member was sought [49]. The appointment of CP Hutchinson, QC as chair of the Royal Commission rapidly followed the completion of his commission of inquiry into Oakley Hospital. Another member of the Royal Commission, J Turnbull, had served on the Royal Commissions on State Services (1961–62) and Social Security (1969–2). Turnbull's appointment may also have been intended to appease psychiatric nurses, because he was a retired national secretary of the Public Service Association. The Royal Commission's membership was criticised for including 'a director of Watties [a major food processing business] and ... a National Party City Councillor' but no nurse, as was first envisaged.[50] The sole medical practitioner on the Royal Commission was not a psychiatrist.

The warrant gave the Royal Commission 28 months to complete its tasks, as befitted the wide mandate of such a prestigious body. In all probability, that time frame would have been unmanageable because of the clumsy three-stage reporting framework. The Royal Commission was asked first to investigate a highly specific industrial issue, then to study psychiatric services, and finally to study the wider hospital system. The possible complications of an approach that moved from the specific to the general than *vice versa *were foreseen but not changed.[51] The Royal Commission received some 90 submissions. Nearly all came from the mental health and health establishment.

The Royal Commission completed a report on stage one but was only part way through stage two when it was wound up prematurely in 1973. The warrant of dissolution cited 'certain events' which had happened after the Royal Commission was appointed, a cryptic allusion to the change of government in late 1972, that was the real reason [52]. The incoming Labour Minister of Health, RJ Tizard, took the view that governments should be proactive and not forestall action by setting up inquiries.[53] The new government wanted to implement its own ideas through a closed-system of considering departmental submissions to its caucus health committee. Abolishing the inquiry suited top departmental officials. They were irritated by the uncomfortable relationship with the Royal Commission, its seeming information overload, and by the way the inquiry's proceedings were hogged by one party with an antagonistic attitude towards the Department.[54]

The Royal Commission completed the first part of its investigation on the 'mental health lead' or differential pay scale that favoured psychiatric and psychopaedic (intellectual handicap) hospital nurses over general hospital nurses. The Royal Commission recommended continued payment of the lead by way of allowance but that psychiatric hospital staffing should be improved to eliminate the need for the differential. The report also called for a study of various factors that affected the recruitment and retention of nursing staff in psychiatric hospitals, such as poor working conditions or the entry criteria for male nursing students.

Just before it was abolished, the Royal Commission published two more but very slender reports. The second was termed 'an immediate interim report', and recommended the setting up of a national post-graduate institute of psychiatry to boost the recruitment and retention of psychiatrists.[55] The third report concerned provision for mentally handicapped persons. The third report concerned provision for mentally handicapped persons. The Royal Commission's criticisms of existing model of care were shown in recommendations for a moratorium on the capital development of psychopaedic hospitals, for a national survey of psychopaedic hospital patients, for the development of a range of alternative general hospital and community based facilities, and for the transfer of administrative responsibility for mental handicap within government from health to social welfare. The very brevity of this third and 'final' report shows that it must have been prepared from a preliminary draft report on the same subject.[56] Neither report did justice to the 'comprehensive report' on mental health services envisaged for stage two of the inquiry.

The Department was embarrassed by all of the Royal Commission's reports. The first report cut across departmental arguments for salary parity. Officials therefore advised that the lead should be retained only as an interim measure, pending efforts to align entry standards for nurse training and conditions of employment. Independent investigation of conditions at selected general and psychiatric hospitals was not favoured.[57] By the time the Royal Commission's other reports were considered, the incoming Labour government had set up its caucus health committee. That committee was a convenient mechanism to massage the official response to the Royal Commission's thinking.[58,59] The Department contemptuously dismissed the proposed institute of psychiatry as a 'grave risk of putting the clock back' and sought solutions within general post-graduate medical education. The proposal itself was referred to key agencies, but their opposition was entirely predictable [60]. Faint praise lavished on the report on mental handicap services as a superficially 'clear, simple and logical answer' masked caution about the impractical staffing implications. By the time the official response was released, the caucus committee had already endorsed the department's proposals for a moratorium on capital development and for a national survey that effectively forestalled action on 12 of the 20 recommendations of the third report. The Royal Commission's other ideas were to be given further consideration, particularly if they threatened current departmental responsibilities.[61]

### Ministerial Committee of Inquiry (1995–6)

By the 1990s, the heyday of the psychiatric hospital was long past. Deinstitutionalisation was well underway in an era of cost cutting. In its wake, public anxieties about poor co-ordination among multiple service providers were aroused by tragedies and led to some calls to bring back traditional institutions. After police fatally shot an armed man who had a history of mental illness in 1995, a Labour Opposition Member of Parliament prepared a private member's Bill to establish a full-scale inquiry. The government contemplated a 'national task force' to address criticisms, but the Prime Minister ruled out a 'big' inquiry because it would only delay systemic improvements. After another similar shooting, however, the government swiftly ordered its own ministerial inquiry rather than have one imposed by the Opposition through Parliament.[62,63] If improved service co-ordination and public safety were the systemic issues for the inquiry in 1995, there was also an element of political damage control.

The Ministerial Inquiry was the first to acknowledge the country's bicultural heritage. The Chair was Judge KH Mason, who has Ngäi Tahu tribal affiliations. He was also an experienced inquiry chair, having conducted two previous mental health inquiries. One of these, the Committee of Inquiry into Procedures Used in Certain Psychiatric Hospitals (1987–88), drew attention to the prominence of Maori in the statistics of mental illness. The same inquiry had other Maori members, a Maori secretary, and a kaumatua (elder). But the composition of the 1995 inquiry differed from its predecessors in other ways. It had the smallest membership (three) – the other members being a woman lawyer and a senior field worker from a major voluntary mental health service provider. It was the only inquiry without a medical member. The Ministerial Inquiry was unusual, too, in that its office was not based in the capital city.

The Ministerial Inquiry blended formal written procedures, informal discussions with selected parties, and site visits in New Zealand and Victoria. Like the other inquiries, this one undertook no independent scientific research. Of the 720 submissions received, more than 400 came from individuals. Three times the number of submissions was received than expected. Although it was granted an extension of time, the Ministerial Inquiry complained that members had less time to consider the large volume of submissions than they would have wished.[64] The traditional policy community was still represented among these submissions, but a very large number came from consumer, self-help, and support groups, and the Mental Health Foundation as a general advocate for mental health. There was also a distinct Maori voice. The explosion of interest can probably be attributed to Mason's earlier inquiry of 1987–8, which had been enjoined to 'adopt procedures that encourage people to participate in your proceedings'. Hearings therefore took place at hospitals and prisons and on marae (traditional Maori meeting grounds).[65]

The Ministerial Inquiry produced the largest report of the five inquiries. Virtually half of it consists of direct quotations from testimony and submissions. In presenting a very human face to the problems of the mental health services, the Inquiry made 'no apology for not substituting our words for those of the submitters – that would be tantamount to sanitising the objectivity of the submissions.'[66] Mindful, perhaps, of the weight of 81 substantive recommendations made by Mason's 1987–88 inquiry, the Ministerial Inquiry made only five formal recommendations, though many suggestions were essentially recommendations. These few formal recommendations were held to be 'significant in their potential impact'. The inquiry refrained from making 'a raft' of detailed recommendations lest that be interpreted as some indicator of efficiency and unless there was a real expectation that they would be implemented.'[67]

The government's response must have confirmed the Ministerial Inquiry's suspicions about the lack of bureaucratic and political will for change. The form of the recommendations was retained, but not necessarily their substance. Funding, for instance, was increased by $142.2 M. above current levels, but nearly $60 M. had to be found from within existing budgets. The government established the Mental Health Commission as a 'tightly-focussed' body with a 'watch-dog role', not as the quasi-department envisaged by the Inquiry. The most likely explanation is that the recommended body would have cut across the prevailing management ideology of the policy-purchaser-provider split. Just as important, it would have reduced the Ministry's own claim to national leadership. The Minister of Health, Jenny Shipley (1993–96), was satisfied that doubling the staff within the ministry's mental health group would 'significantly boost' the ministry's performance in providing policy advice and monitoring services.[68] The ministry's policy leadership role was enshrined in the same legislation that set up the Mental Health Commission.[69] Far from the Minister's confident expectation that members of the inquiry would be satisfied by the government's response, they quickly and contemptuously condemned the bureaucratic meddling.[70]

## Discussion

The five New Zealand general inquiries into mental health have been undertaken about once a generation, or the same frequency of general stock-takes of mental health legislation. That pattern matches Simpson's findings on public inquiries in other policy domains.[71] Two explanations might account for the infrequency of systemic stock-takes. First, mental health policy serves multifaceted social purposes of control, care and cure. Policy is complex and it can be controversial because numbers of mental disordered people are subject to involuntary detention, assessment and treatment – the most commonplace use of such powers in health legislation. Protecting fairly the civil liberties and safety of the mentally disordered individual, families and caregivers, and the public involve difficult medico-legal decisions. Ideology asserts a powerful influence upon policy solutions. Mental health lacks high status and popularity. Fickle public attitudes vary from sympathy to stigma and stereotypes. Politicians prefer not to disturb the basics of mental health policy. TF Gill, Minister of Health (1975–8), suggested that 'the broad problems of mental health' can readily become matters for rather pointless political controversy' unless skilfully handled. He saw little merit in tying such issues to specific political programmes.[72]

The nature of public policy-making and government funding may also explain why advisory inquiries have been held so infrequently. RJ Polaschek, a distinguished New Zealand public servant, saw government as largely incremental, with periodic bursts of activity when marginal adjustments fell short of public expectations.[73] Mental health policy development in New Zealand, as elsewhere, has been characterised by spasmodic bursts of reform interspersed with long periods of stagnation or neglect.[74-76] Major initiatives, general reviews of legislation or services, significant organizational changes, and significant injections of new funding mark booms in the saga of New Zealand's national mental health policy [77]. The corresponding low points include the end of either a long period of continuous government by one party, or the incumbency of long-serving top officials.

The circumstances that surrounded the formation and follow-up of every inquiry involved a mix of policy issue and politics. Each inquiry was formed when a systemic policy issue reached a level of political sensitivity and public significance. An inquiry – or the threat of one – has created a 'climate for action' as Chapman puts it.[78] Prasser has suggested that setting up a public inquiry may serve several political purposes, including some that seem relevant to the five mental health inquiries studied here. According to Prasser, a public inquiry may elicit more specific information to guide the government. It may help to define policy problems more precisely or more acceptably at the political level. An inquiry may provide a broader range of policy options than might emerge from the public service. An independent inquiry is a way to impartially review existing arrangements, to resolve public controversy, or to promote public participation and consensus. Prasser considers that an inquiry can help a government to manage the policy agenda by the illusion of action, deflection of criticism, or co-option of critics.[79]

The authority, mandate, composition, procedure and reporting style of the five inquiries reflect trends among public inquiries generally. Parliament initiated the two nineteenth century inquiries; the twentieth century inquiries were instruments of the executive, a trend consistent with the constitutional realignment in the state. The main forms of inquiry are represented in the mental health line-up, which points to the flexibility of the device. All inquiries except the Ministerial Inquiry were based in the capital city of the day.

Membership trends in the five inquiries are consistent with those in inquiries generally. Each twentieth century inquiry was chaired by a legal practitioner. AW Mackay suggests that in Canada this convention creates 'instant credibility and an aura of objectivity and independence'.[80] The size of inquiry teams has shrunk, although New Zealand inquiries have generally been small by British standards.[81] The odd number of members in most cases may have been intended to safeguard unanimity, as Simpson contended.[82] Eminent citizens were chosen in different combinations of regional, gender, professional/lay and ethnic perspectives. It is interesting to note how the number of medical practitioners has dropped except for the Board of Health Committee, which was a technical body. Sir Keith Holyoake's views, which were stated earlier, reveal a 'romantic yearning for the commonsense approach to the solution of social problems and a profound distrust of professional expertise', as Borchardt suggested in his analysis of inquiries in New South Wales.[83] The Royal Commission and Ministerial Inquiry best reflected what AR Prest termed the Noah's ark principle or the trade-off of expertise, representativeness and official acceptability.[84]

Each inquiry has considered and arbitrated among different perspectives and shaped its thinking according to the weight of evidence. Inquiries before 1957 consulted only 'interested parties', or the small circle of officials, agencies, professionals, and industrial or professional associations directly involved in providing psychiatric care. Such selective involvement has been superseded by the notion that a public inquiry is an exercise in participative democracy. Any interested organisation or person who responds to a public advertisement can make a submission. Increased public awareness and the proliferation of mental health interest groups in recent years, thanks to administrative devolution, deinstitutionalisation, and state sector restructuring, is illustrated by the exponential growth in the number of submissions made to the Ministerial Inquiry from submissions received by the earlier inquiries.

The formality of proceedings has slowly been relaxed, although all inquiries have kept a verbatim record or minutes of proceedings. Since the 1960s, inquiries have followed a process of considering written submissions followed up by public hearings, cross-examination by other parties, and questioning by the inquiry team.[85] The Ministerial Inquiry blended formal written procedures, informal discussions with selected parties, and site visits in New Zealand and Victoria.[86] None of the five inquiries undertook an independent scientific research programme.

All reports were unanimous, although the Board of Health Committee narrowly averted a majority report. This fact bears out Weller's observation that inquiries have a choice of working entirely in the open (above stage), negotiating (behind stage) or doing secret deals (below stage).[87] Inquiries completed their task fairly quickly, although timing was a problem in two cases. The truncation of the Royal Commission was unique in New Zealand administrative history, though apparently not in the United Kingdom.[88]

An inquiry's 'sole legacy' is its report which, Hallett suggested, is said to emit 'a more or less musty aroma.'[89] Each inquiry presented a single report at the conclusion of its proceedings, save for the Royal Commission, with three reports. As a matter of custom rather than law, each inquiry's report was published soon after it was presented. By contrast with twentieth century reports, the select and Joint Committee reports were remarkably concise. The lengthier reports of twentieth century inquiries have summarised and analysed the evidence in order to support the findings and recommendations. The Ministerial Inquiry produced the largest report of the five bodies.

Three general themes haunt the reports of the five mental health inquiries. A self-evident wish to improve standards of care and treatment points firstly to the limited importance of mental health over time in the overall priorities of government. Intermittent public interest is insufficient to resolve ongoing resource problems of adequate funding, specialist staffing, and proper facilities. The Minister of Health, Jenny Shipley, said as much when she released the report of the Ministerial Inquiry. 'Governments come and go, ministers come and go – we've had a 20-to-25 year problem where mental health has always been left last,' she said.[90] The problem is actually far older, as New Zealand's Inspector-General of Lunatic Asylums reported in 1898:

The public are very exacting in their demands for the proper treatment of the insane, but they are roused to indignant clamour only when some painful occurrence reveals the difficulties which their officers are daily confronted with and almost despairingly struggle to overcome. In the intervals, there is no sustained resolve that their representatives shall provide the means of proper classification and treatment.[91]

The role of the specialised institution in the care and treatment of mental disorder is another recurrent theme. The nineteenth century inquiries sought properly equipped lunatic asylum(s). The twentieth century inquiries faced the growing limitations of that investment in institutional psychiatry. Adopting a less institutional approach, they have recognised the need for effective co-ordination among a growing range of services. The third theme concerns the need for an effective national organisation and accountability framework to provide clear direction and leadership. This theme was most obvious in the inquiries of 1871 and 1995–96.

Prest rightly suggests that an inquiry has the difficult task of aiming its report somewhere between the rock of a politically appealing set of recommendations and the hard place of publication and damnation.[92] Salter explains the same problem as the contradiction in a process whereby an inquiry can incorporate quite radical debate as well as quite limited, highly pragmatic and reformist goals of producing specific policy recommendations.[93] The place of an inquiry's report on that continuum of specificity depends upon several factors. For instance, inquiries typically do not constrain their thinking within specified resource limits, so the financial cost of recommendations may be too great. The political cost of unusual or radical thinking may mean rejection, delay, or further investigation. Moreover, changes in the political environment between the inception and report of an inquiry can significantly affect the outcome. Electoral misfortune limited the effectiveness of the inquiries of 1858 and 1972–3. Cabinet reshuffles, another potentially disruptive political factor, did not disturb the other inquiries, which completed their work under the same minister.

Bureaucratic influence, however, has exerted a powerful mediating role. The nineteenth century inquiries encountered no national mental health bureaucracy, but the three twentieth century inquiries show how the department of state has acted to protect or promote its own interests. Mutualism between ministers and officials is intrinsic to Westminster constitutional systems and the Whitehall/Wellington administrative systems, which may help explain why the Department's long record in managing mental hospitals was not scrutinised by an inquiry more often. Almost certainly such scrutiny would have subjected administrators and their ministers to embarrassment. The Royal Commission's hearings on psychiatric services took place shortly after the transfer of mental hospitals, a technicality that enabled departmental officials to focus on the possibilities of the post-transfer environment rather than the deficiencies of the past.

Departmental archives demonstrate the multiple roles of the bureaucracy throughout the life of the Board of Health Committee and the Royal Commission. Officials recommended the establishment of the inquiry, drafted terms of reference and nominated expert members – all activities that fall within the normal range of advice to ministers. The department provided the committee's secretariat and that of the Royal Commission, again in line with accepted practice.[94] The Ministerial Inquiry declined a similar offer. The department provided basic factual information, made submissions and gave evidence to each inquiry since 1957. Inquiries have also provided a chance outside the usual lines of accountability for officials to state their concerns and to make their suggestions. The late Dr DP Kennedy, Director-General of Health (1965–72), told me at the time the Royal Commission was set up, that an inquiry was one of the few occasions when a department could fly its own kites.

Bureaucratic mediation, however, has been strongest in advising ministers how to respond to an inquiry's report. As ad hoc instruments of public administration, they play no part in implementing their own ideas, a point that the Ministerial Inquiry (and an earlier inquiry chaired by Judge Mason) may not have understood. Their reports proposed mechanisms to implement recommendations, which indicated a lack of confidence in the department.[95,96] Inquiries lack the organisational continuity, institutional memory, technical expertise, and political influence of a permanent departmental administration. Bureaucratic influence is considerable in determining the fate of recommendations when an inquiry's vision converges with resource realism and political endorsement. Table 1 shows a trail of discarded or severely modified recommendations that met a political and a financial cost or priority too high to pay. This has often been as a major weakness of the inquiry as an instrument of public policymaking.[97-101]

Assessing the overall effectiveness of the five public inquiries by the popular criterion of immediate implementation of a report's recommendations is problematic. Such a unidimensional standard raises three important questions that can be answered by examples from the five inquiries. Does immediate mean a usable time when the report of an inquiry retains some currency among decision-makers? Literal interpretation of 'immediate implementation' distorts the record of each inquiry. The 1858 inquiry, for instance, would score highly as considerable progress was made towards implementing the basic recommendation about a general lunatic asylum. But the foundation stone of such an institution was never laid let alone its roof. Two years and a change of government later, the practicality of building a national facility virtually disappeared. Similarly, account should be taken of the vacillation of the governments between 1871–4 in implementing the substantive recommendation of the Joint Committee to appoint a national inspector. The recommendation was ultimately but not immediately implemented. The staffing arrangements proposed for regional psychiatric units by the Board of Health Committee were initially implemented but abandoned only a few years later.

Next, does implementation refer to the letter or the spirit of recommendations? The capital moratorium imposed by the Department far exceeded the Royal Commission's recommendation. The form of the recommended Mental Health Commission was retained in the follow-up to the Ministerial Inquiry, but not the substance.

The third question is whether the standard of immediate implementation should apply to all the recommendations or just the key ones. Not all recommendations are equal and they can not be judged equally. Some recommendations, however, were vague, sweeping, and had few handles for implementation, for example:

That whilst steps should be taken to improve all Asylums of the Colony, the state of that at Karori, near Wellington, urgently requires immediate attention and reform.[102]

A dissemination of knowledge of the various types of mental illness should lead to an early and more accurate diagnosis of mental illness by general practitioners. Such a general understanding, it is felt, may even prevent the onset of mental illness and contribute to the maintenance of good mental health....[103]

The specificity of some recommendations, however, like many of those of the Royal Commission, may lend themselves more easily to immediate implementation. Yet any list is likely to contain both key and derivative or secondary recommendations. An assessment of the Royal Commission's work is also hampered by the fact that it did not have the opportunity to complete its mandate.

The durable impact on mental health policy in New Zealand of the core ideas of each report is considerable. For example, the parliamentary inquiries promoted the concept of the ideal lunatic asylum. The ideal was never attained in the concept of a single national institution, but the same ideals underpinned the establishment of a network of provincial asylums. The romance and nostalgia of the ideal asylum could still be found in officialdom more than a century later.[104] The 1858 inquiry also paved the way for a comprehensive and stand-alone mental health statute. That principle has been followed in every general review of mental health legislation from the Lunatics Act 1868 to the present Mental Health (Compulsory Assessment and Treatment) Act 1992. The third example involves a specialised organisation within the machinery of central government that would, *inter alia*, advise ministers on policy, and monitor plans and policies. The Joint Committee mooted that idea and the Ministerial Inquiry upheld its importance. Fourth, two inquiries made a major contribution to the modern policy of deinstitutionalisation. The Board of Health Committee report expedited the provision of acute psychiatric services outside of mental hospitals. The Royal Commission's report on mental handicap services led to the moratorium on capital development in all mental hospitals and to a national survey of all patients in mental hospitals that was intended to identify the need for alternative community based services.

These durable effects fit what Le Dain terms the social function of public inquiries which, he claims, is probably more important in the long term than specific recommendations:

What gives an inquiry ... its social function is that it becomes, whether it likes it or not, part of this ongoing social process. There is action and interaction.... Thus this instrument ... may have a dimension which passes beyond the political process into the social sphere. The phenomenon is changing even whole the inquiry is in progress. The decision to institute an inquiry of this kind is a decision not only to release an investigative technique but a form of social influence as well.[105]

The social role of inquiries has helped to widen the mental health policy community far beyond the original 'establishment'. Participation in the proceedings of an inquiry may engender a sense of social contribution to the solution of problems. An investigatory inquiry can be publicly cathartic.

## Conclusion

Public inquiries are an accepted tool in the process of public policy-making. They have played a direct and indirect role in shaping mental health policy throughout the history of government in New Zealand and beyond that to the British parliamentary inquiries of the eighteenth and early nineteenth centuries. Although their utility will no doubt continue to be debated, history shows that the public inquiry – in both its advisory and investigative forms – remains an important tool of public administration.

The reports provide valuable snapshots of the general state of New Zealand's mental health services at different stages of evolution. The effectiveness of any inquiry should not be judged solely by the immediate acceptability of its report. The cause-effect relationship may take much longer. The report of an inquiry may be prophetic but contribute to a climate of opinion that supports subsequent change, maybe years later, long after any direct causal link could be determined. Although implementation of an inquiry's findings and recommendations is important in assessing its effectiveness, if we consider the long-term impact of the core ideas of each report, the five inquiries can be linked to several significant and long-term contributions to mental health policy in New Zealand.

## Abbreviations

*AJHR Appendix to the Journals of the House of Representatives*. Wellington: New Zealand Parliament 1854-.

Ministerial Inquiry Inquiry under Section 47 of the Health and Disability Services Act 1993 in Respect of Certain Mental Health Services.

*NZPD New Zealand Parliamentary Debates*. Wellington: New Zealand Parliament 1854-.

## Competing interests

The author(s) declare that they have no competing interests.

## Authors' contributions

I am the sole author of this paper.
